# Supplementary figures and images for: VIRGO2: Unveiling the Functional and Ecological Complexity of the Vaginal Microbiome with an Enhanced Non-Redundant Gene Catalog
Source: bioRxiv. 2025 Mar 4:2025.03.04.641479. Preprint. [Version 1] doi: 10.1101/2025.03.04.641479 (PMC11908257; doi:10.1101/2025.03.04.641479)

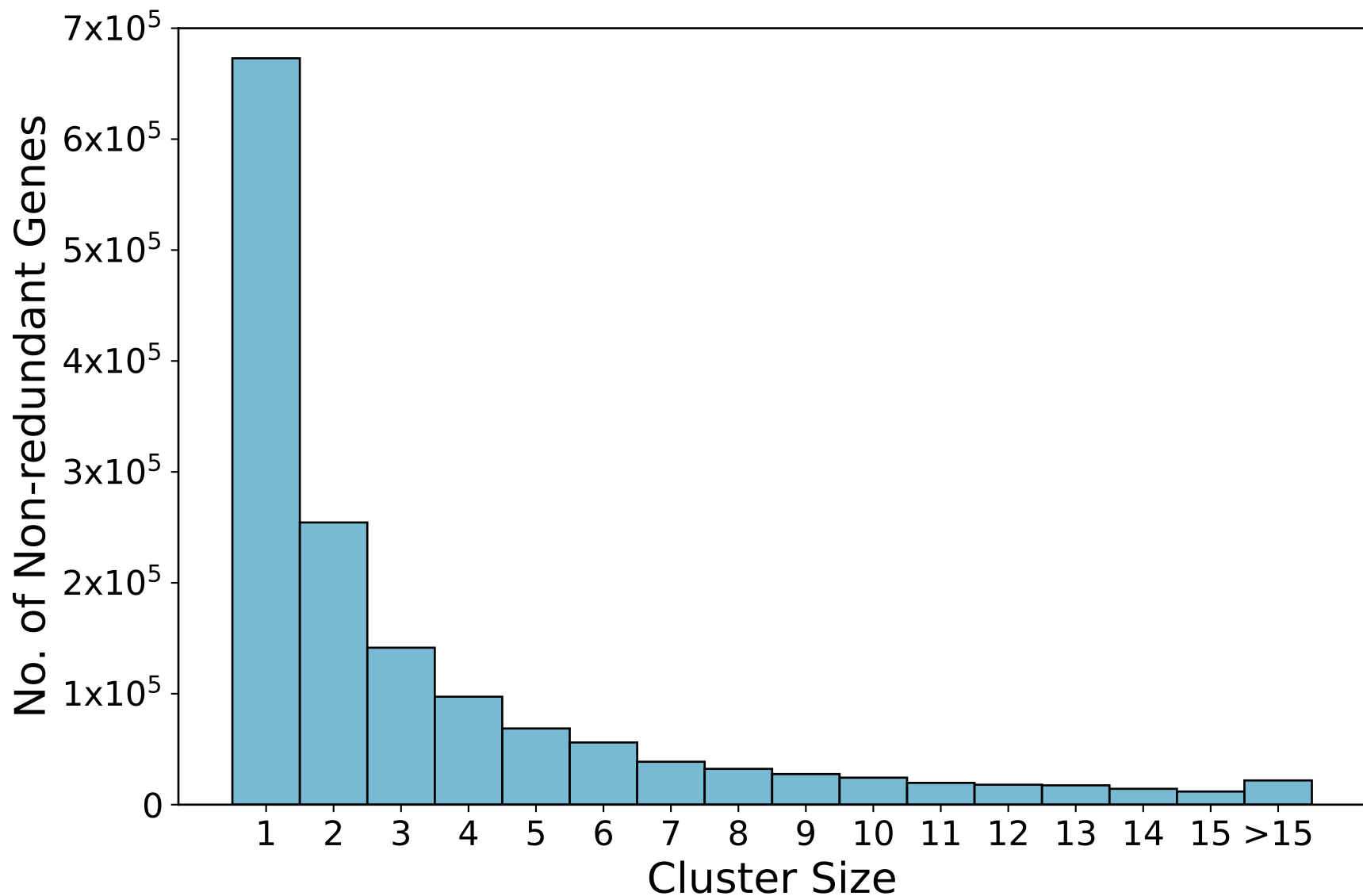

Supplement: Supplement 1 — Supplementary Figure S1: Histogram displaying the number of non-redundant genes by the size of the cluster [file media-1.pdf]

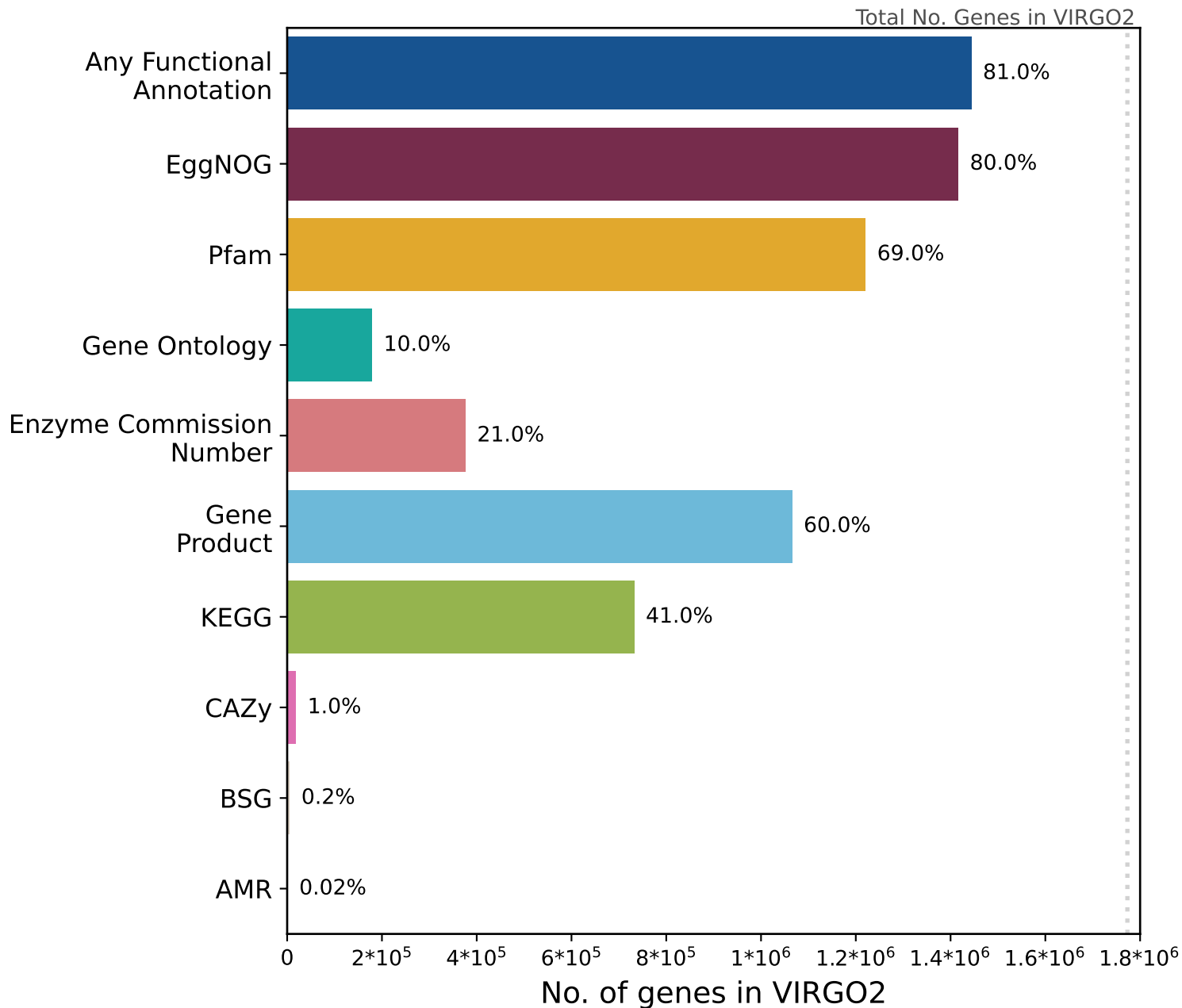

Supplement: Supplement 2 — Supplementary Figure S2: Percent of VIRGO2 non-redundant genes assigned to various functional annotation schemes [file media-2.pdf]

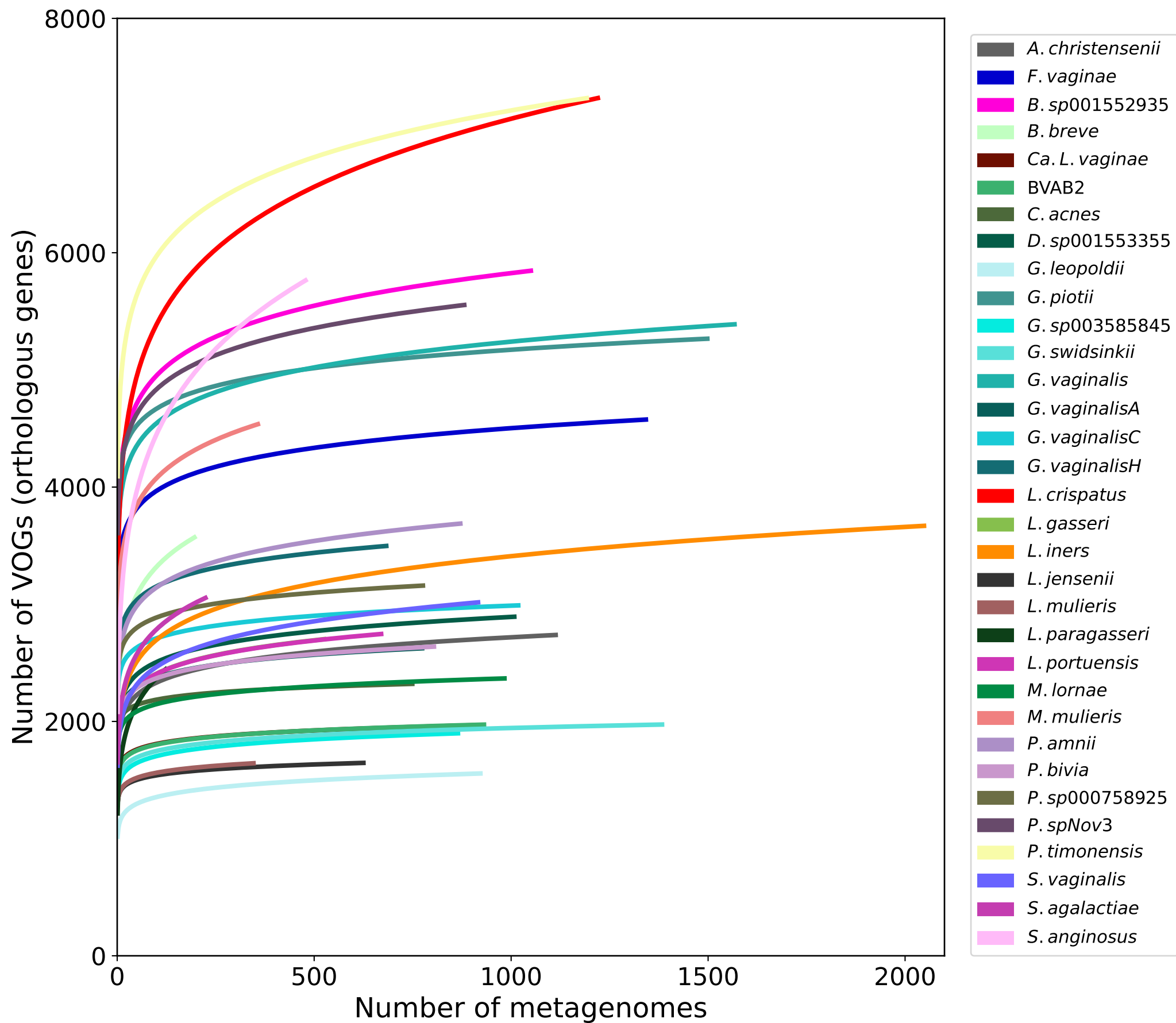

Supplement: Supplement 3 — Supplementary Figure S3: Metagenome accumulation curves of prominent vaginal bacteria assessed at the level of orthologs (VOGs) [file media-3.pdf]

ROC for prediction of CST IV-A, IV-B

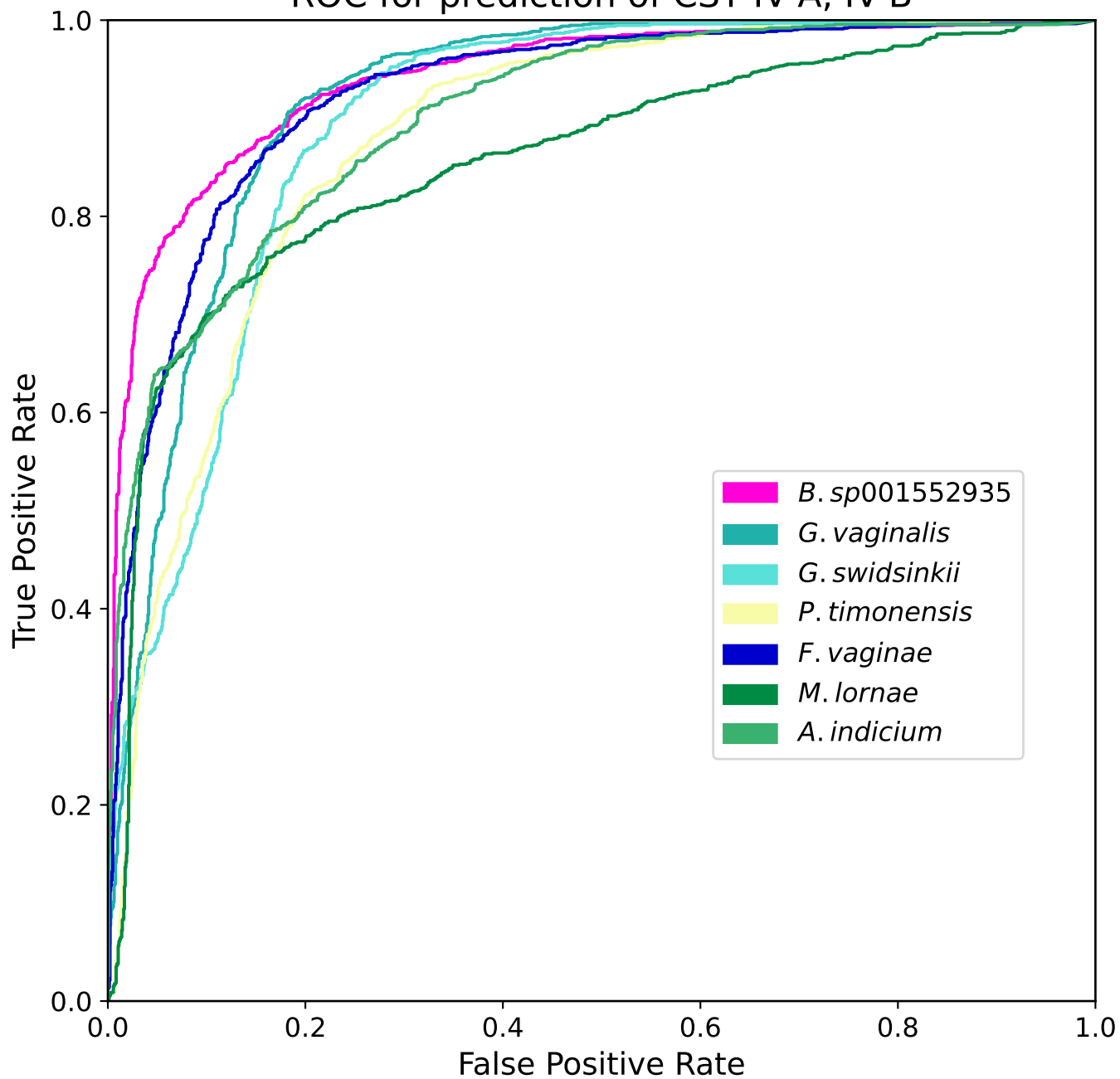

Supplement: Supplement 4 — Supplementary Figure S4: Receiver operating characteristic curves demonstrating the ability of the relative abundances of select microbial species (Berryella sp001552935, G. vaginalis, G. swidsinskii, P. timonensis, F. vaginae, M. lornae, and A. indicium) to predict an overall community composition assigned to CST IV-A or IV-B. [file media-4.pdf]
