# Supplementary material for: VIRGO2: Unveiling the Functional and Ecological Complexity of the Vaginal Microbiome with an Enhanced Non-Redundant Gene Catalog
Source: bioRxiv. 2025 Mar 4:2025.03.04.641479. Preprint. [Version 1] doi: 10.1101/2025.03.04.641479 (PMC11908257; doi:10.1101/2025.03.04.641479)
Supplement: Supplement 6 — Additional File 2: Original, unaltered TEM micrographs used in Figure 5. [file media-6.pdf]

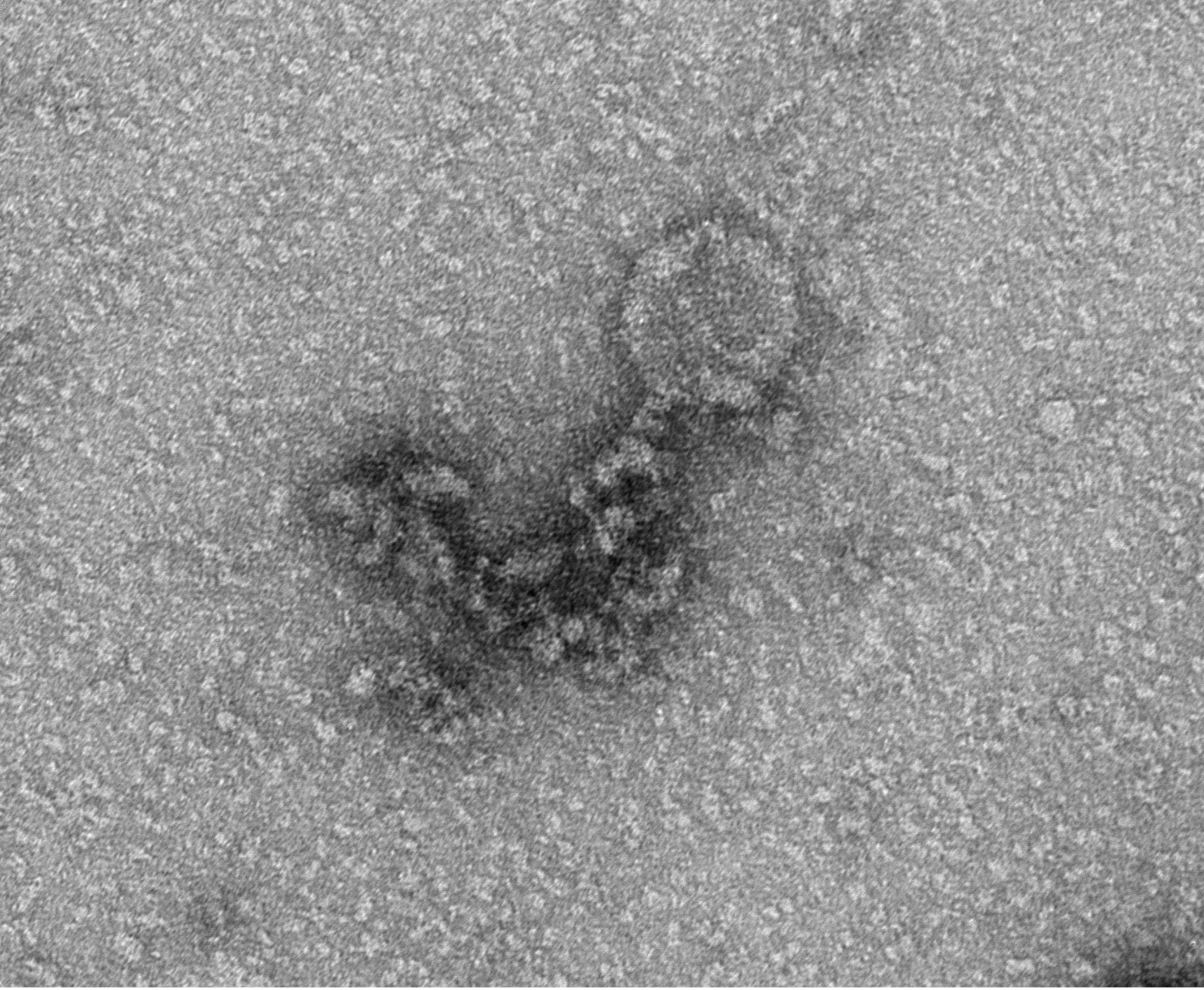

X-54_TEM1-PHG1_S1_009.tif
X-54_Ravel
TEM1-PHG1_S1
Cal: 0.145603 nm/pix
9:38:52 a 05/30/24
TEM Mode: Imaging
Microscopist: MLD

20 nm
HV=80.0kV
Direct Mag: 67000x
UMB EMCIF

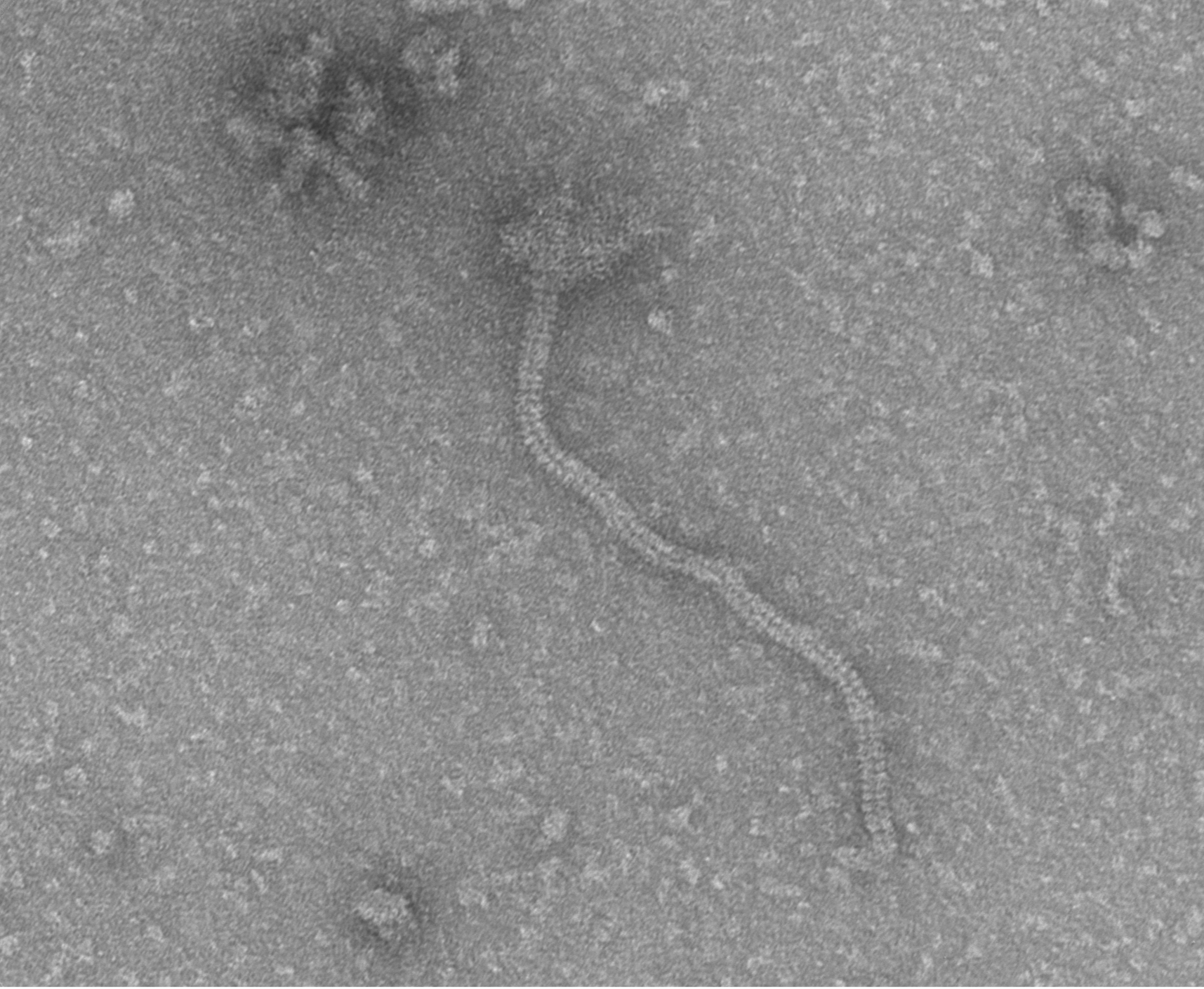

X-54_TEM1-PHG1_S1_032.tif
X-54_Ravel
TEM1-PHG1_S1
Cal: 0.145603 nm/pix
10:27:25 a 05/30/24
TEM Mode: Imaging
Microscopist: MLD

20 nm
HV=80.0kV
Direct Mag: 67000x
UMB EMCIF
